# Association of psoriasis with geographic and fissured tongue in the Han population in southwestern China^☆^

**DOI:** 10.1016/j.abd.2024.01.006

**Published:** 2024-08-06

**Authors:** Yuting Hu, Ying Li, Wei Yan, Yu Zhou

**Affiliations:** a State Key Laboratory of Oral Diseases, National Center for Stomatology, National Clinical Research Center for Oral Diseases, Research Unit of Oral Carcinogenesis and Management, Chinese Academy of Medical Sciences, West China Hospital of Stomatology, Sichuan University, Chengdu, Sichuan, China; b Department of Dermatology, West China Hospital, Sichuan University, Chengdu, Sichuan, China

**Keywords:** Case-control Study, Glossitis, benign migratory, Mouth mucosa, Psoriasis, Tongue, fissured

## Abstract

**Background:**

Psoriasis is a common immune-mediated skin disease that can involve other organs and tissues, including the oral mucosa. Some studies have found an increased proportion of geographic tongue (GT) and fissured tongue (FT) in patients with psoriasis, which appears to be region-specific.

**Objectives:**

The association of psoriasis with GT/FT in Eastern Asian populations remains unknown. Thus, the authors aimed to investigate the association of psoriasis with GT/FT in the Han population in southwestern China.

**Methods:**

This study was conducted on 230 psoriatics and 230 healthy controls at West China Hospital. The authors compared the proportion of subjects with GT/FT in the two groups and compared age, gender, smoking, alcohol consumption, age at onset of psoriasis, duration of psoriasis, nail and joint involvement, Psoriasis Area and Severity Index, Body Surface Area, Dermatology Life Quality Index, and proportion using biologics in psoriatics with or without GT /FT.

**Results:**

The authors have found a strong association between psoriasis and FT (*p* < 0.001), and a non-significant association between psoriasis and GT (*p* = 0.760). Compared to psoriasis patients without FT, the authors found that psoriasis patients with FT were older (*p* = 0.021) and had an increased frequency of late-onset psoriasis (*p* = 0.014); they also had more severe psoriasis (*p* = 0.047) and poorer quality of life (*p* = 0.045).

**Study limitations:**

GT has periods of exacerbation and remission, so the authors cannot avoid a deviation of the prevalence of GT in this study from the true prevalence rate. Also, biologics have been found to lead to remission of GT and FT, which may have influenced the GT/FT ratio in the case group in this study.

**Conclusions:**

Psoriasis was associated with FT in the Han population in southwestern China, attention must be paid to the treatment of psoriatics with FT and skin diseases in patients with FT.

## Introduction

Psoriasis is a common immune-mediated, chronic inflammatory skin disease, in which genetic factors play a key role. However, the importance of environmental factors, such as smoking and alcohol consumption, is increasing.^1^, ^2^ Its prevalence is approximately 2%.^3^ Limited or widespread scaly red patches are its typical manifestation, which can be classified as common, pustular, erythrodermic, and arthritic depending on the clinical lesion. In addition to the skin, it can also involve the fingernails/toenails and joints, seriously affecting patients’ quality of life. Psoriasis can affect the oral mucosa and is known as “oral psoriasis”, with many studies in recent years focusing on its non-specific oral lesions: Geographic Tongue (GT) and Fissured Tongue (FT).^4^

GT is a benign, chronic, recurrent inflammatory disorder of the tongue, which is characterized by multifocal erythematous patches formed by atrophy of the filiform papillae, surrounded by a slightly elevated white or yellow border formed by hyperplasia of the filiform papillae, creating a map-like appearance. The lesions are variable in pattern and location and are known as “migratory glossitis” accordingly. Its prevalence is 0.28%‒14.4%. It is most common in children and declines with age, women are more frequently affected than men.^5^ Risk factors for GT include immunological, genetic, allergic, and psychological factors, it may be accompanied by other diseases, such as psoriasis, FT, burning mouth syndrome, diabetes mellitus, and Down's syndrome.^6^ GT is usually asymptomatic, but patients often develop serious anxiety and fear of cancer, thus a detailed explanation of this disease is needed. Some patients experience burning, and discomfort sensations, symptomatic treatment may be effective, such as antihistamines, anxiolytics, and steroids.^5^

FT is usually believed a congenital anomaly and characterized by fissures on the dorsal and/or dorsolateral aspects of the tongue in different arrangements and depths.^7^ The incidence is 0.6%‒29.2%,^8^ its frequency increases with age. The etiology of FT is also unknown, genetic factors may be a major risk factor for FT. FT often coexists with GT, Down's syndrome, acromegaly, Sjogren's syndrome, and Melkersson-Rosenthal syndrome.^7^

The prevalence of psoriasis, GT, and FT are all regionally specific.^8^ The association of psoriasis with GT/FT in East Asian populations remains unknown. Therefore, this study aims to determine the association of psoriasis with GT/FT in the Han population in southwest China and characterize these associations in terms of patients’ general condition, condition of psoriasis, and the treatment modality.

## Methods

This cross-sectional, case-control study was designed and conducted in accordance with the World Medical Association Declaration of Helsinki. This study was approved by the local ethics committee. All 460 subjects had given informed consent. 230 patients with psoriasis treated at the Department of Dermatology, West China Hospital, Sichuan University from October 2022 until February 2023 were included in the case group, this study did not exclude patients with psoriasis using any treatment including biologics. 230 healthy subjects accompanying other patients, never diagnosed with psoriasis and matched for age and sex to the case group, were included in the control group.

The diagnosis of psoriasis was performed by a professional dermatologist and based on typical clinical manifestations: scaly red plaques, confirmed by histopathological examination if the lesions are atypical. All subjects underwent a detailed oral examination. The oral examination was performed by qualified dentists experienced in the diagnosis and treatment of oral diseases using stomatoscopes and swabs under artificial light. GT was diagnosed by its typical clinical presentation: atrophy of the filiform papillae as a red plaque-like lesion with hyperplasia of the surrounding filiform papillae. GT is a chronic relapsing-remitting disease with periods of exacerbation and remission, so typical clinical photographs of GT were prepared to determine whether each subject had this lesion previously. FT is also diagnosed by its typical clinical manifestations: fissures on the dorsal and/or dorsolateral aspects of the tongue in different arrangements and depths.

General information including age, sex, smoking, alcohol consumption, age at first onset of psoriasis (early-onset: onset before or at the age of 40, late-onset: onset after 40 years), duration of psoriasis, nails and joints involvement, Psoriasis Area and Severity Index (PASI), Body Surface Area (BSA), Dermatology Life Quality Index (DLQI), and the type of treatment received are recorded.

The authors compared the proportion of subjects with GT and FT in the case and control groups and compared age, sex, smoking, alcohol consumption, age at first onset of psoriasis, duration of psoriasis, nails and joints involvement, PASI, BSA, DLQI, and proportion using biologics in psoriatic with or without GT/FT.

Data were analyzed using the IBM SPSS Statistics 26 version. According to the results of the Kolmogorov-Smirnov test, Continuous variables in this study were shown as median (interquartile range) and were compared using the Mann-Whitney *U* test. Categorical variables were shown as numbers (percentage) and were compared using Pearson's Chi-Square test or Fisher's exact test. A two-sided *p*-value less than 0.05 was considered to be statistically significant.

## Results

460 subjects were included in this study, including 230 patients with psoriasis and 230 healthy controls. The case group consisted of 136 males and 94 females with a median age of 36 (29‒49). The control group consisted of 123 males and 107 females with a median age of 39 (31‒51) (Table 1). The case and control groups were matched for age and sex (age: *p* = 0.130, sex: *p* = 0.222).

**Table 1:** General characteristics of case and control group.

Total (n = 460)	Number of patients (%)/Median (IQR)		Z/χ^2^ value	*p*-value
	Psoriasis (n = 230)	Controls (n = 230)		
Age, years	36 (29‒49)	39 (31‒51)	−1.513	0.130
Sex				
Male	136 (59.1)	123 (53.5)	1.493	0.222
Female	94 (40.9)	107 (46.5)		

IQR, Interquartile Range.

The authors have found a non-significant association between psoriasis and GT (*p* = 0.760) and a strong association between psoriasis and FT (*p* < 0.001) (Table 2).

**Table 2:** GT and FT in case (n = 230) and control group (n = 230).

	Number of patients (%)		χ^2^ value	*p*-value
	Psoriasis (n = 230)	Controls (n = 230)		
GT				
Yes	5 (2.2)	6 (2.6)	0.093	0.760
No	225 (97.8)	224 (97.4)		
FT				
Yes	37 (16.1)	5 (2.2)	26.831	<0.001^a^
No	193 (83.9)	225 (97.8)		

FT, Fissured Tongue; GT, Geographic Tongue.

a p < 0.001, A significant statistical difference was observed.

No differences were observed between psoriasis patients with GT and those without GT in terms of Age, sex, smoking, alcohol consumption, age at first onset of psoriasis, duration of psoriasis, nails and joints involvement, PASI, BSA, DLQI, and proportion using biologics. Compared to psoriasis patients without FT, the authors found that psoriasis patients with FT were older (*p* = 0.021) and had an increased frequency of late-onset psoriasis (*p* = 0.014), they also had more severe psoriasis (*p* = 0.047) and poorer quality of life (*p* = 0.045). No associations of FT with other characteristics, including sex, smoking, alcohol consumption, duration of psoriasis, nails and joint involvement, BSA, or proportion using biologics, were observed in the case group (Table 3).

**Table 3:** Associations of GT/FT with clinical characteristics in 230 psoriasis patients.

	Number of patients (%)/Median (IQR)		Z/χ^2^ value	*p*-value	Number of patients (%)/Median (IQR)		Z/χ^2^ value	*p*-value
	GT (n = 5)	No GT (n = 225)			FT (n = 37)	No FT (n = 193)		
Age, years	32 (31‒35)	36 (29‒49)	−0.765	0.444	41 (30‒55)	35 (29‒45)	−2.311	0.021^a^
Sex								
Male	1 (20)	135 (60)	3.238	0.162	27 (73)	109 (56.5)	3.496	0.062
Female	4 (80)	90 (40)			10(27)	84 (43.5)		
Smoking								
Yes	0 (0)	63 (28)	1.928	0.326	8 (21.6)	55 (28.5)	0.738	0.390
No	5 (100)	162 (72)			29 (78.4)	138 (71.5)		
Alcohol consumption								
Yes	0 (0)	28 (12.4)	0.708	>0.990	6 (16.2)	22 (11.4)	0.674	0.414
No	5 (100)	197 (87.6)			31 (83.8)	171 (88.6)		
Onset								
Early	5 (100)	188 (83.6)	0.980	>0.990	26 (70.3)	167 (86.5)	6.080	0.014^a^
Late	0 (0)	37 (16.4)			11 (29.7)	26 (13.5)		
Duration, years	6 (1‒13)	10 (3‒17)	−0.513	0.546	10 (4‒20)	10 (3‒15)	−0.371	0.710
Nail psoriasis								
Yes	1 (20)	10 (4.4)	2.599	0.219	2 (5.4)	9 (4.7)	0.038	0.692
No	4 (80)	215(95.6)			35 (94.6)	184 (95.3)		
Psoriatic arthritis								
Yes	0 (0)	19 (8.4)	0.46	>0.990	2 (5.4)	17 (8.8)	0.474	0.746
No	5 (100)	206 (91.6)			35 (94.6)	176 (91.2)		
PASI	1.3 (0‒3.9)	1.5 (0.5‒4)	−0.287	0.774	2.7 (1‒5.6)	1.4 (0.5‒3.9)	−1.987	0.047^a^
BSA	2 (0‒4)	1 (0.5‒4)	−0.100	0.920	2 (1‒6)	1 (0.5‒3)	−1.896	0.058
DLQI	8 (2‒9)	5 (2‒10)	−0.379	0.705	7 (2‒13)	4 (1‒9)	−2.005	0.045^a^
Biologics								
Yes	3 (60)	95 (42.2)	0.632	0.653	15 (40.5)	83 (43)	0.077	0.781
No	2 (40)	130 (57.8)			22 (59.5)	110 (57)		

FT, Fissured Tongue; GT, Geographic Tongue; IQR, Interquartile Range.

a p < 0.05, A significant statistical difference was observed.

## Discussion

Psoriasis is a common, chronic, immune-mediated disease, it is not an isolated skin disease and is associated with an increased risk of comorbidities including psoriatic arthritis, cardiovascular disease, diabetes mellitus, and gastrointestinal disorders.^9^ It is also thought to involve the oral mucosa, known as “oral psoriasis”. The first case of oral psoriasis was reported in 1903,^10^ and subsequently, several cases have been reported.^11^, ^12^, ^13^ In these articles, the manifestations of oral psoriasis can be divided into two groups: (1) Psoriasis-specific lesions, and (2) Non-specific lesions.^14^ The psoriasis-specific lesions are highly heterogeneous and can present as diffuse erythema of the palate, histopathological examination shows a psoriatic pattern (hyperkeratosis, acanthosis with downward elongation of the rete ridges, mixed infiltration with neutrophils and lymphocytes, congested blood vessel, Munro abscesses).^12^ It can also manifest as whitish and erythematous plaques/papules on the buccal mucosa.^13^ When it occurs on the lip, it can appear as a dry, flaky lip, similar to cheilitis.^11^ The presentation on the gingiva is similar to desquamative gingivitis.^12^ Oral non-specific lesions in psoriatic include GT and FT, which occur not only in psoriasis but also in other diseases. In recent years, many case-control studies have found an increased proportion of GT and FT in patients with psoriasis, but still, some studies have not observed statistically significant differences. The association between GT/FT and psoriasis remains controversial.

Many epidemiological studies reports have found a correlation between psoriasis and GT.^15^, ^16^, ^17^, ^18^, ^19^, ^20^, ^21^, ^22^ Psoriasis and GT share a common genetic marker-human leukocyte antigen (HLA-Cw6).^23^ Nevertheless, psoriasis and GT have similar histopathological features: parakeratosis, acanthosis, Kogoj microabsces, Munro microabscesse, vascular ectasia and inflammatory cell infiltration (particularly T-lymphocytes and neutrophils) in the submucosa.^24^ However, no statistically significant association was found between GT and psoriasis in this study (*p* = 0.760), probably because of the regional specificity of the prevalence of GT, it was low in both the case group (2.2%) and control group (2.6%) in this study, which is below the levels already reported.^15^, ^16^ GT is more prevalent in females and children. However, in psoriasis patients, some studies have found that GT occurs more frequently in men^15^ and older subjects,^18^ which is not found in this study (age: *p* = 0.444, sex: *p* = 0.162). Some studies have shown a higher frequency of alcohol consumption in patients with psoriasis or GT,^14^ and studies have also shown that smokers have a lower frequency of GT, which may be attributed to the fact that nicotine activates the nicotinic acetylcholine receptors on macrophages, thus leading to a reduction in the synthesis of TNF-α, IL-1 and IL-6,^25^ these immune cells and cytokines are also important in psoriasis. So environmental factors such as smoking, and alcohol consumption may be involved in the development of GT in psoriasis. Interestingly, none of the five psoriasis patients with GT in this study smoked or drank alcohol, although the differences were also not statistically significant (smoking: *p* = 0.326, alcohol drinking: *p* > 0.990). Picciani et al. have found that GT was more frequent in early-onset psoriasis and is associated with disease severity. Early-onset psoriasis is generally more severe than late-onset psoriasis and is more associated with HLA-Cw6,^26^ so they suggest that GT could be an indicator of psoriasis severity.^21^ Darwazeh et al. have found that the proportion of GT is higher in psoriasis patients whose quality of life is significantly affected.^27^ Unlike the article above, in this study, the prevalence of GT in the case group was not related to the condition of psoriasis, such as age at first onset of psoriasis (*p* > 0.990), duration of psoriasis (*p* = 0.546), nails (*p* = 0.219) and joints (*p* > 0.990) involvement, PASI (*p* = 0.774), BSA (*p* = 0.920), DLQI (*p* = 0.705). In the present study, a higher proportion of use of biologics was observed in psoriasis patients with GT compared with psoriasis patients without GT, but the difference was not statistically significant (*p* = 0.653). A study has found remission of GT in psoriasis patients treated with secukinumab.^28^ Overall, this study did not find any statistically significant association between psoriasis and GT.

This study found a significant correlation between FT and psoriasis (*p* < 0.001), consistent with several studies.^15^, ^16^, ^17^, ^18^, ^19^, ^20^, ^21^, ^29^, ^30^, ^31^ FT was significantly associated with the age of patients with psoriasis (*p* = 0.021) and age at the first onset of psoriasis (*p* = 0.014); Compared to psoriasis patients without FT, the authors found that psoriasis patients with FT were older and had an increased frequency of late-onset psoriasis, which is in line with published studies,^16^, ^30^ this may be explained partly by an increased prevalence of FT with age. Gonzaga et al. found a lack of association between FT and HLA-Cw6, suggesting that they did not find a common genetic background for FT and psoriasis.^32^ Thus, FT is regarded as a continuation of GT by some authors,^4^, ^14^ which can partly explain the association between psoriasis and FT found in this study. The authors have found a significant association between severity of psoriasis and FT (PASI: *p* = 0.047), which is in line with Altemir et al. and contrary to Picciani et al.,^30^, ^31^ Picciani et al. also report the clinical differences in the FT of patients with and without psoriasis, they found the branching and diffused pattern of FT with moderate to severe scale was more frequent in patients with FT and psoriasis. The authors observed that psoriasis patients with FT had worse quality of life (*p* = 0.045), compared to psoriasis patients without FT, it may be due to more severe manifestations of psoriasis as well as oral manifestations of FT.

This study still has limitations; GT has periods of exacerbation and remission, and although typical clinical photographs of GT were prepared to determine whether each subject had this lesion previously, the authors cannot avoid the deviation of the prevalence of GT in this study from the true prevalence rate, so prospective cohort studies with larger sample sizes are warranted. Also, biologics have been found to lead to remission of GT and FT,^28^, ^33^ which may have influenced the GT/FT ratio in the case group in this study.

The prevalence of psoriasis, GT, and FT are all regionally specific.^8^ Most of the case-control studies on the association of psoriasis with GT and FT have been conducted in Europe, southeastern Asia, western Asia, southern Asia, North America, and South America (Table 4), which have a different racial composition compared with East Asia, and there is no relevant evidence of the association of psoriasis with GT and FT in East Asian populations, so this is the first case-control study on the correlation between psoriasis and GT/FT in Han population in southwest China. These findings are similar to those of Monshi et al. and Altemir et al.,^29^, ^31^ the authors have shown that psoriasis is significantly correlated with FT, and FT is associated with the age of patients with psoriasis, age at first onset of psoriasis, severity of psoriasis, and patients' quality of life in the case group. No statistically significant association was found between psoriasis and GT in this study. Therefore, attention needs to be paid to the treatment of psoriatics with concomitant FT, as well as to the documentation and follow-up observation of skin diseases in patients with FT.

**Table 4:** Case-control studies investigating the association of psoriasis with GT/FT in different countries.

Country	Psoriasis/Controls	Psoriasis with G/Controls with GT (*p*-value)	Psoriasis with FT/Controls with FT (*p*-value)	Reference
Iran	200/200	28/12 (<0.012)	66/19 (<0.0001)	17
Mexico	80/127	10/6 (0.0447)	38/26 (<0.0001)	18
Brazil	166/166	30/7 (0.0001)	57/27 (0.0002)	16
Liban	400/1000	31/10 (<0.0001)	133/99 (<0.0001)	19
Italy	535/436	49/23 (0.032)	121/45 (<0.01)	20
Jordan	100/100	17/9 (0.09)	35/13 (0.000)	27
India	600/800	34/7 (<0.0001)	272/320 (0.0456)	15
Brazil	348/348	43/10 (0.002)	125/70 (<0.001)	21
Brazil	129/5871	21/399 (<0.05)	NA^a^	22
Austria	173/173	4/4 (1)	25/13 (0.04)	29
Spain	100/100	4/2 (0.68)	39/16 (<0.001)	31

a NA, Not Available, FT is not covered in this article.

## Financial support

This work was supported by the Fund of Sichuan Provincial Department of Science and Technology (2022YFS0039), the 10.13039/501100001809National Natural Science Foundation of China (82370974, 81771086), the Research and Develop Program, West China Hospital of Stomatology, Sichuan University (LCYJ2023-DL-2), the CAMS Innovation Fund for Medical Sciences (CIFMS, 2019-I2M-5-004).

## Authors’ contributions

Yuting Hu: Data collection, analysis, and interpretation; statistical analysis; writing of the manuscript; final approval of the final version of the manuscript.

Ying Li: Critical review of the literature; final approval of the final version of the manuscript.

Wei Yan: The study concept and design; effective participation in the research guidance; final approval of the final version of the manuscript.

Yu Zhou: The study concept and design; effective participation in the research guidance; final approval of the final version of the manuscript.

## Conflicts of interest

None declared.
